# Beliefs and attitudes towards participating in genetic research – a population based cross-sectional study

**DOI:** 10.1186/1471-2458-13-114

**Published:** 2013-02-07

**Authors:** Samantha M Kerath, Gila Klein, Marlena Kern, Iuliana Shapira, Jennifer Witthuhn, Nicole Norohna, Myriam Kline, Farisha Baksh, Peter Gregersen, Emanuela Taioli

**Affiliations:** 1 Hofstra-North Shore School of Medicine, 175 Community Drive, Great Neck, NY 11021, USA; 2 Feinstein Institute for Medical Research, North Shore- Long Island Jewish Health System, 350 Community Drive, Great Neck, NY, 11021, USA; 3 Monter Cancer Center, North Shore- Long Island Jewish Health System, 450 Lakeville Road, Lake Success, NY, 11042, USA; 4 North Shore- Long Island Jewish Health System, 175 Community Drive, Great Neck, NY 11021, USA

**Keywords:** Biological specimen banks, Genetic research, Public opinion

## Abstract

**Background:**

Biobanks have the potential to offer a venue for chronic disease biomarker discovery, which would allow for disease early detection and for identification of carriers of a certain predictor biomarker. To assess the general attitudes towards genetic research and participation in biobanks in the Long Island/Queens area of New York, and what factors would predict a positive view of such research, participants from the NSLIJ hospital system were surveyed.

**Methods:**

Participants were recruited at six hospital centers in the NSLIJ system during the summers of 2009 and again in 2011 (n = 1,041). Those who opted to participate were given a questionnaire containing 22 questions assessing demographics, lifestyle and attitudes towards genetic research. These questions addressed individual participant’s beliefs about the importance of genetic research, willingness to participate in genetic research themselves, and their views on informed consent issues.

**Results:**

Respondents took a generally positive view of genetic research in general, as well as their own participation in such research. Those with reservations were most likely to cite concerns over the privacy of their medical and genetic information. Those who were married tended to view genetic research as important, while those in the younger age group viewed it as less important. Prior blood donation of respondents was found to be a predictor of their approval for genetic research. Demographic factors were not found to be predictive of personal willingness to participate in genetic research, or of approval for the opt-out approach to consent.

**Conclusions:**

While respondents were generally inclined to approve of genetic research, and those who disapproved did not do so based on an underlying moral objection to such research, there is a disconnect between the belief in the importance of genetic research and the willingness of individuals to participate themselves. This indicates a continued concern for the ways in which genetic materials are safeguarded once they are collected. Also indicated was a general lack of understanding about the various consent processes that go along with genetic research, which should be addressed further to ensure the successful continuation of biobanks.

## Background

Biobanks are the repository of a large number of individuals’ biological samples, annotated with clinical and/or genetic information. The utilization of data and samples stored in existing biobanks has become the cornerstone of human genome and epigenome research. The information is utilized, for example, to understand how environmental factors can effect genome expression as it relates to health and disease occurrence [1,2]. Biobanks have the potential to offer a venue for chronic disease biomarker discovery, which would allow for disease early detection and for identification of carriers of a certain predictor biomarker. This could eventually enable subjects to undertake preventive measures to decrease their individual disease risk [3-10].

Despite its potential benefits, genetic research is a highly sensitive topic for investigators, the public, patients and ethicists alike, as genetic data is encoded in the human tissue and is potentially identifiable. Single nucleotide polymorphisms (SNPs) and other germ-line genetic traits are shared with siblings, as well as other family members, and inherited from parents. An individual’s ethnicity, geographic birthplace, as well as many other inherited characteristics may be inferred from the acquisition of information in a person’s SNPs [11]. This can have implications beyond the subject’s donation to the biobank; it may affect the individual’s family, and even his/her racial or ethnic group affiliation [12].

An individual can be identified within the large set of public data already existent in the published genome- wide association study, using only small subsets of an individual’s genome [13]. As noted by Lin et al., a unique individual can be identified with as few as 30–80 SNPs [14]. Although information about an individual donor is not immediately apparent, theoretically, with considerable effort and economical resources, said subject could be identified. This can be achieved through comparison with genetic information obtained from a personal item, or comparison to DNA from a relative [15], combined with available information from separate databases, such as the National Geographic’s Genographic Project, as well as specimens collected from people serving in the military [16] and arrested for criminal offenses [17].

While there are currently several hundred biobanks throughout the world, storing several million samples, many social issues have been raised surrounding the creation and maintenance of such biobanks, including our own. Issues such as personal and societal benefits, informed consent, enrollment of vulnerable populations, confidentiality, privacy, benefit of sharing, intellectual property, data access, returning results to participants, and commercial exploitation remain critical for maintaining the public trust and for volunteers’ continued participation in biobanks [16].

Very few studies have addressed population beliefs and attitudes towards genetic research and biobanking. The purpose of the current study is to understand the general population’s attitudes towards consenting and banking of genetic material. To accomplish this aim, an anonymous survey was conducted during the summer of 2009 and replicated in 2011 among North Shore Long Island Health System (NSLIJHS) patients and their families with the aim of assessing how people and patients feel about banking their DNA for research purposes and about the prerequisite consent process for inclusion in genetic research.

## Methods

### Questionnaire development

After reviewing other validated surveys [18-20], a condensed, 22 question version was created by our research team, with targeted multiple choice questions and several open-ended, qualitative questions. A health literacy expert was involved as a consultant to ensure that the questions fit at least an 8th grade reading level. The 2009 questionnaire consisted of three sections- one that addressed beliefs towards genetic research, one that addressed questions of personal privacy, and one on the process of obtaining consent for genetic research. The format was compatible with the Scantron grading system (Scantron Corporation, Eagan MN). An additional section consisting of 4 questions was added to the 2011 questionnaire, and was aimed at assessing attitudes toward returning individual research results from genetic testing to study participants.

### Survey administration

**Figure 1:**
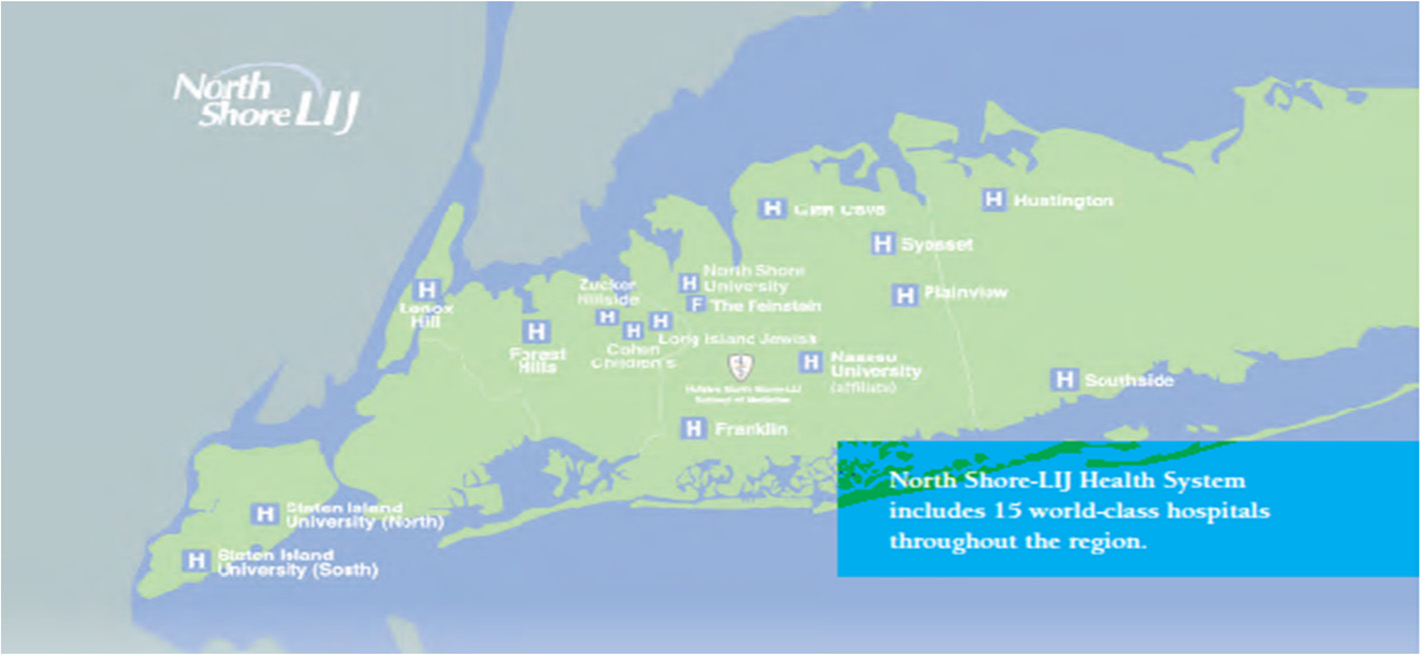
Map of Long Island and Staten Island with the NSLIJHS hospitals.

Two anonymous surveys were conducted in 2009 and in 2011 as part of the Institutional commitment to periodically monitor the interest of the population in community-based genetic research. A questionnaire on attitudes towards genetic research and banking of genetic material was distributed to a convenience sample of subjects attending various facilities within NSLIJHS, representing the diverse geographic, socioeconomic and ethnic catchment areas of the Health System. NSLIHS is a network of 15 hospitals (Figure 1) that covers the population of Nassau, Suffolk and Queens County in Long Island, and Richmond County (Staten Island). The following six centers within the NSLIJHS were targeted: Center for Advanced Medicine, New Hyde Park; Monter Cancer Center, New Hyde Park; Franklin Hospital, Valley Stream; North Shore Ambulatory Surgery, Manhasset; Southside Hospital, Bay Shore; Plainview Hospital, Plainview. Preliminary meetings were conducted with the Directors of the various sites and permission was obtained to conduct the survey in patient waiting rooms.

Recruitment was performed by trained interviewers who approached patients and families in the NSHLIJHS waiting rooms and asked if they were willing to answer an anonymous questionnaire for research purposes.

The project underwent IRB expedited approval because of the anonymous nature of the survey through the Feinstein Institute of Medical Research at North Shore LIJHS. Due to survey anonymity, participants would not be able to be re-contacted for further assessment of their attitude toward genetic research. In 2009, 523 people answered the questionnaire; in 2011 there were 518 respondents, for a total population of 1,041 participants.

### Statistical analysis

Data are reported as mean +/− SD and percentages. Comparisons between means were performed using the t-test; comparison of percentages was done using the chi-square test. Multiple logistic regression was run to assess the determinants of a positive response to genetic research and participation in a biobank (Questions 1, 2, 3, and 7). For this analysis, the responses to the two surveys (2009 and 2011) were pooled. Data were analyzed using SPSS software (SPSS Inc., Chigaco ILL).

## Results

The characteristics of the population included in this survey are reported in Table 1. Overall, the two samples were very similar in terms of demographics. Most of the respondents were female (63.7%), married (63.6%) and in the median 40–59 age group (46.1%). About half of the remaining respondents were split between the younger (18–39 years, 24.8%) and the older age group (60+ years, 29.2%). Most of the respondents were White (68.1%), with the second largest represented ethnic group being Black (13.4%). The remaining 18.6% was distributed fairly equally between Hispanic (7.2%), Asian (6.3%) and Other (5.1%), which encompassed those of multiracial, American Native and Pacific Islander ancestry. A large majority of respondents had some form of higher education (79.3%), with 26.1% representing those with graduate degrees, and 53.2% with at least some college education.

**Table 1:** Characteristics of the respondents to the two surveys

**Variable**	**N = 1,041 N (%)**
**Age (years)**	
18-29	125 (11.94)
30-39	134 (12.82)
40-49	209 (19.98)
50-59	273 (26.11)
60-69	185 (17.7)
70-79	87 (8.31)
80+	33 (3.16)
**Gender**	
Male	378 (36.33)
Female	663 (63.68)
**Ethnicity**	
White	708 (68)
Black	139 (13.36)
Hispanic	75 (7.23)
Asian	66 (6.34)
Other	53 (5.09)
**Highest education attained**	
Some HS	39 (3.76)
Completed HS	176 (16.9)
Some college	275 (26.4)
Bachelor degree	279 (26.80)
Graduate degree	272 (26.15)
**Marital status**	
Single	224 (21.34)
Married	667 (63.61)
Separated/divorced	106 (10.11)
Widowed	52 (4.96)
**Religion**	
Protestant	116 (11.30)
Catholic	513 (49.96)
Jewish	171 (16.66)
Other	159 (15.48)
None	69 (6.62)
**Have children**	
Yes	771 (74.87)
No	259 (25.14)
**Major illness in 1**^**st**^**degree relative**	
Yes	608 (58.74)
No	395 (38.17)
Don’t know	32 (3.1)
**Ever donated blood**	
Yes	556 ( 53.43)
No	399 (38.32)
Tried, but I was not accepted	86 (8.23)

As shown in Table 2, four target questions aimed at assessing individual perception of genetic research were also included. Question 1 referred to the respondent’s opinion of societal importance of genetic research, Question 2 assessed personal approval of genetic research, Question 3 addressed use of respondent’s own genetic data for research purposes, and Question 7 addressed respondent’s attitudes towards the idea of an “opt-out” of consent policy for inclusion in genetic studies*.* The large majority (97.7%) of respondents said “yes” or “maybe” to the idea that it is a “gift” to society when an individual takes part in medical research. The remaining 2.3% of participants did not find taking part in medical research to be important gift to society (Question 1).

**Table 2:** Distribution of responses in the two surveys

**Question**	**N =1,041 N (%)**
**Beliefs about Genetic Research**	
1. Do you feel it is an important gift to society when a person takes part in medical research	
Yes	885 (83.02)
Maybe	156 (14.64)
No	25 (2.35)
2. How do you feel about genetic research	
Approve	875 (82.24)
Unsure	180 (16.91)
Disapprove	9 (0.86)
**3. How would you feel about your genetic data being taken from you blood sample (your identity would not be known) and being used in medical research**	
**Approve**	751 (70.79)
**Unsure**	253 (23.84)
	57 (5.37))
**Personal privacy**	
4.Would you be willing to join the BioGeneBank *	
Yes	556 (52.72)
Not sure	364 (34.36)
No	137 (12.94)
5. Reasons for refusal among those who answered not sure/no:	
Having an extra tube of blood drawn	66 (14.22)
Keeping information private and secure	332 (74.02)
Interfering with nature by taking part in genetic res.	49 (11.6)
6. Would you be willing to provide extra info: about your daily habits	
Yes	586 ( 56.95)
Most likely	275 (26.68)
May be	122 (11.83)
No	47 (4.54)
6a. about your family’s health	
Yes	682 (66.38)
Most likely	211 ( 20.5)
May be	89 (8.66)
No	46 (4.46)
**Informed consent process**	
7. Would you be comfortable if an “opt-out” approach was used in the BioGene Bank?	
Yes	461 (44.37)
Most likely	197 (18.96)
Maybe	166 (15.97)
No	215 (20.71)
8a. Uncomfortable enrolling in a research without having the study explained to^#^	
Yes	376 (61.94)
Probably	122 (20.11)
May be	64 (10.52)
No	46 (7.43)
8b. Concerned about being enrolled in a research without my knowledge^#^	
Yes	459 (74.77)
Most likely	83 (13.48)
May be	39 (6.44)
No	33 (5.33)
8c. Worried that other kinds of research would be done that I don’t approve of^#^	
Yes	384 (62.18)
Probably	89 (14.42)
May be	76 (12.45)
No	68 (10.96)

*this question was formulated slightly differently in the 2011 questionnaire; the statement “in the future” was added to the question.

^#^excluding those who answered ‘yes’.

Respondents were also very approving of genetic research in general (Question 2), with 82.2% unequivocally in favor of genetic research, and only .8% against such studies. Positive responses were similarly high regarding Question 3, although the approval of use of one’s own genetic material was slightly lower, with a disapproval rate of 5.7%. A large majority of those who disapproved of their own genetic information being used in research responded as such out of concern for the security of their personal information (75.3%), while others were concerned about having an extra tube of blood drawn (14.3%), and interfering with nature through their participation (10.3%).

Positive responses toward the use of an “opt-out” of consent policy (Question 7) for inclusion in genetic research were far lower than those regarding the research itself. Results were pooled for clarity of analysis, with 36.7% of respondents answering No/Maybe, as opposed to only 63.3% of respondents who answered “Yes/Probably”

### Multivariate analysis

The four questions assessing attitudes towards genetic research (Questions 1, 2, 3 and 7) were analyzed. The demographic factors that best explained results for the target questions on attitudes towards genetic research were Age and Marital Status of respondent. General attitudes towards societal importance of genetic research were significantly associated with Age and Marital status (Table 3). Younger subjects were less likely to respond “Yes/Maybe” to the question (Odds Ratio: 0.21; 95% CI = 0.04-0.97). Participants who were married, however, were more likely to view genetic research as important for public benefit (Odds Ratio: 5.14; 95% CI = 1.18-22.35) than subjects who were not.

**Table 3:** Predictors of respondent’s belief in societal importance of genetic research (multivariate regression analysis)

***Variable***		***Odds of responding “YES” to Question 1***	***95% Confidence Intervals***
*Age(years)*	18-39	0.21	0.04-0.97
	40-59	0.38	0.10-1.42
	60+	Ref	
*Gender*	Male vs. Female	1.44	0.57-3.62
*Ethnicity*	White vs. Other	1.23	0.43-3.53
*Education Level*	≤ HS Diploma	0.75	0.23-2.45
	≤ College Diploma	1.94	0.67-5.53
	Graduate Degree	Ref	
*Marital Status*	Married vs. Single/Divorced/ Separated/Widowed	5.14	1.183-22.35
*Being a parent*	Yes vs. No	0.96	0.24-3.77
*Major Illness in 1*^*st*^*Degree Relative*	Yes vs. No/don’t know	0.55	0.22-1.40
*Blood Donation*	Yes/Tried vs. No	0.80	0.32-2.01
*Religion*	Yes vs. No	1.47	0.43-4.03

When assessing general approval of genetic research (Table 4), having donated blood in the past was a predictor of approval of genetic research (Odds Ratio: 1.83; CI = 1.28-2.60). The individual willingness to participate in genetic research (Table 5) was not influenced by age, gender, race or education. No demographic factor, including ethnicity and religion, was significantly associated with approval of the use of the “opt-out” consent process in genetic research (Table 6).

**Table 4:** Predictors of respondent’s approval of genetic research (multivariate regression analysis)

***Variable***		***Odds of responding “Approve” to Question 2***	***95% Confidence Intervals***
*Age (years)*	18-39	1.09	0.63-1.89
	40-59	1.10	0.71-1.70
	60+	Ref	
*Gender*	Male vs. Female	1.00	0.69-1.46
*Ethnicity*	White vs. Other	1.33	0.90-1.97
*Education Level*	≤ HS Diploma	0.92	0.55-1.53
	≤ College Diploma	0.87	0.57-1.33
	Graduate Degree	Ref	
*Marital Status*	Married vs. Single/Divorced/Separated/Widowed	1.12	0.74-1.71
*Being a parent*	Yes vs. No	1.20	0.74-1.94
*Major Illness in 1*^*st*^*Degree Relative*	Yes vs. No/I don’t know	1.12	0.78-1.60
*Blood Donation*	Yes/Tried vs. No	1.83	1.28-2.60
*Religion*	Yes vs. No	0.91	0.64-2.86

**Table 5:** Predictors of respondent’s willingness to personally participate in genetic research (multivariate analysis)

***Variable***		***Odds of responding “YES” to Question 3***	***95% Confidence Intervals***
*Age (years)*	18-39	0.75	0.30-1.85
	40-59	0.76	0.38-1.50
	60+	Ref	
*Gender*	Male vs. Female	0.84	0.45-1.58
*Ethnicity*	White vs. Other	1.04	0.53-2.06
*Education Level*	≤ HS Diploma	0.52	0.22-1.24
	≤ College Diploma	0.58	0.30-1.12
	Graduate Degree	Ref	
*Marital Status*	Married vs. Single/Divorced/ Separated/Widowed	1.12	0.54-2.29
*Being a parent*	Yes vs. No	0.65	0.27-1.58
*Major Illness in 1*^*st*^*Degree Relative*	Yes vs. No/I don’t know	0.92	0.49-1.71
*Blood Donation*	Yes/Tried vs. No	0.98	0.38-2.66
*Religion*	Yes vs. No	0.89	0.39-1.58

**Table 6:** Predictors of respondent’s attitudes towards opt-out consent (multivariate analysis)

***Variable***		***Odds of answering “YES” to Question 7***	***95% Confidence Intervals***
*Age (years)*	18-39	0.68	0.44-1.05
	40-59	0.75	0.53-1.05
	60+	Ref	
*Gender*	Male vs. Female	0.86	0.64-1.15
*Ethnicity*	White vs. Other	1.02	0.74-1.41
*Education Level*	≤ HS Diploma	1.03	0.69-1.54
	≤ College Diploma	1.27	0.92-1.74
	Graduate Degree	Ref	
*Marital Status*	Married vs. Single/Divorced/ Separated/Widowed	0.85	0.61-1.18
*Being a parent*	Yes vs. No	1.05	0.71-1.54
*Major Illness in 1*^*st*^*Degree Relative*	Yes vs. No/don’t know	0.82	0.62-1.09
*Blood Donation*	Yes/Tried vs. No	0.79	0.60-1.05
*Religion*	Yes vs. No	0.78	0.62-1.53

## Discussion

In this survey the majority of the respondents were supportive of genetic research, with a very small minority being unequivocally opposed to such studies, whether broadly or on a personal level. The respondents who did not approve of their genetic material being used in research were generally opposed based on issues pertaining either to privacy of their personal information or an unwillingness to have extra blood drawn. Very few were opposed on moral grounds, indicating that a large majority of participants who answered negatively about participating in research do not have an underlying disapproval for this type of research.

Previous research done by the U.S Department of Human Services and the US Department of Labor revealed 63% of responders would not participate in genetic studies due to fear of workplace or insurance discrimination caused by genetic conditions being revealed through participation in genetic testing and/or research [21]. While this federal survey was over a decade ago, more recent studies also show a persistent fear of adverse effects of utilization of genetic data, which underlies many Americans refusal to participate in genetic research [20]. With the passage of the Federal Genetic Information Non-Discrimination Act of 2008, which disallows the request or usage of genetic information in any detrimental way by employers and/ or insurance providers [21], public confidence in the protection of their information has increased, but still remains a very real deterrent. This confidence also revolves around trust in particular institutions and/or experts who are undertaking the research, which can vary greatly by region and by population [22]. The identification of specific participant concerns may lead to future studies on the possibility of enhanced legislative safeguards on personal medical information when participating in genetic research, and of the effects this type of security may have upon future participation.

There have been very few studies which assess the attitudes towards genetic research in the United States, and even fewer on non-selected or hospitalized populations in the Northeast region of the country. This type of research has generally been done on either special populations, such as U.S veterans, or on populations whose demographics do not match our own, such as those from the 2011 Vanderbilt University study, which was based out of Nashville, Tennessee [23]. Studies which have been done in the northeast have concentrated more on ethical questions pertaining to privacy of genetic information, such as that undertaken by researchers at Johns Hopkins University [24].Outside of the U.S, several large scale studies, such as those in Scotland [18], Ireland [19] Canada [25], have been undertaken to assess different population attitudes towards genetic research and biobanking; however, differences in legislative, social and demographic factors, make it difficult to extrapolate the results to the Northeastern U.S.

Divergent from many previous studies was the fact that demographic factors, including race and gender, were not predictors of willingness to participate in genetic research. In this large geographic area, encompassing Queens, Nassau and Suffolk counties, ethnicity of the respondent was not predictive of attitudes toward genetic research. It is interesting that despite the national debate surrounding genetic research, which is highly influenced by religious interest groups, the majority of our respondents did not have any such moral objection; instead, most were not opposed to the research in general, only to its possible personal consequences. The questionnaire responses indicate that nearly all participants, with the exception of a combined 6.6% from both survey years, considered themselves as part of a religious group, yet a very small percentage disagreed with genetic research on moral/religious grounds.

This indicates that perhaps the political and/or social composition of the Queens/Long Island area has an effect which overrides most of the religious objections which are more prevalent in other geographic areas. In many other studies done throughout the United States, racial identity is often a strong predictor of willingness to participate in a research study, with minorities being sometimes less willing to participate due to historical abuses of their participation in such research [26]. In a 2006 study conducted in the Southern United States it was shown that African Americans were more than 20% less likely to enroll in a genetic study [26]. However, this was not the case in our study, where belonging to a specific racial category did not prove predictive of approval for, or willingness to participate in, genetic studies. It is likely that our results are reflective of the reality and beliefs of the most recent years, when education programs on clinical trials launched among minority and underserved communities have continued to shed light on the importance of research in general.

Another important issue highlighted by this survey dealt with the concerns that survey respondents had regarding the use of an “opt-out policy” in obtaining informed consent. In such a policy, patients admitted or treated in any of the NSLIJHS hospitals would be automatically enrolled in the BioGene Bank research study unless they sign a waiver stating that they would like to be excluded. This policy is being utilized increasingly across the country in order to ensure that genetic research programs can continue to enroll subjects and contribute to potentially groundbreaking new studies [27]. Part of the controversy surrounding genetic research in general is the way in which information is obtained, and our results mirror this, with only 44.4% responding that they would definitely be satisfied with the “opt-out” approach for obtaining consent, and 20.7% definitely opposed to the idea of this method. In addition, over 80% of those who responded to this question were uncomfortable with a study being done without a specific explanation, and without their knowledge. The present analysis showed that none of the demographic factors influences approval for the “opt out” of consent policy, and that the issue should be explored further. Yet when analyzing the qualitative responses following this question, it was clear that the “opt out” concept was not clear to the participants, and this may explain the participants’ conflicting responses regarding this topic. Appropriate programs aimed at clarifying what the options are when the “opt out” of consent policy is in place may be necessary in these populations found to be more likely to disapprove of this policy.

While bio-repositories have been in existence for several decades, there is still a paucity of literature on the subject of the “opt-out” consent method, and how its utilization could potentially affect individual participation in such repositories. Approval of such a method is often relatively low, and is also often misunderstood. Results of such studies are often ambiguous in regards to one another, and often have an element of confusion as to participant understanding of certain consent policies. One such study, done at Vanderbilt University, shows this confusion, as 85.6% approved of biobanking genetic samples using the opt-out method, but at the same time held the belief that written permission should be obtained in order for DNA samples to be included [23]. Results were far lower, but perhaps better understood, in another 2011 study, in which 67% of survey respondents favored an opt-in, rather than an opt-out consent approach [28]. Similarly, in another survey conducted at five sites throughout the United States, it was shown that 42% of participants (n = 8,735) preferred that consent be obtained for each new research study that an individual’s DNA is used for, thus precluding the use of an opt-out policy [29]. Results in 2006 Finnish and Swedish studies were similar, with 30% of participants (n = 1,195), and 46% of participants (n = 926), respectively, preferring to have consent obtained with each new research study involving their genetic material [20,30]. However, it remains unclear how much individual participants understand the differing processes of consent, and therefore, if the data collected on approval/disapproval of opt-out is an accurate representation of participant’s attitudes towards it.

In the 2011 survey, a question about the possibility of receiving back the individual results of the genetic testing was added: 62.3% of the participants answered they would like to receive individual research results and only 4% said that they do not want to receive individual results. Yet again, the concept of “returning individual research results” was not very clear to participants; it was clear from the open ended responses that participants put enormous expectations into any test that relates to their genetic material, even when the study is strictly experimental and no clinical importance is attached to a result deriving from such study.

The return of individual research results (IRRs) is another issue that is prevalent in ethical debates surrounding biobanking, and genetic research in general. While some feel that it is a moral obligation on the part of researchers to disclose IRRs to participants, others feel that the focus of such genetic research should be on a population or societal level, and not on an individual level [29]. While the debate continues amongst ethicists, researchers and administrators, several studies have been carried out to assess participant’s attitudes on the subject. Consistent with our results is the fact that in the majority of these studies, participants were very eager to receive IRRs, although the preference as to the content of these IRRs is somewhat disparate. This disparity can be attributed to personal preference, but also to the groups participating in these studies. For example, one study conducted among parents of children with genetic or developmental disorders saw a unanimous desire to have all aspects of their children’s IRRs returned to them, whether actionable or not [31]. This group did, however, recognize that there should be an opportunity for parents, and participants, to choose which results should be disclosed to them. In another study conducted among focus groups across the United States, the great majority of participants would want all genetic results returned to them, while markedly less wanted results on conditions that could not currently be treated [29]. In several of the focus groups, participants made it known that they would not want results that were not at all actionable, marking a divergence from those who are living with those with genetic and/or developmental issues already. Overall, results that could not be interpreted or offered little insight into specific conditions were not highly valued.

This study has several limitations: the study sample may not be representative of certain aspects of the national demographic data since the last census; for example, the percentage of respondents of Hispanic ancestry, is 7.2%, which is far below the national average of 16.3% [32].

Another limitation is the voluntary participation of the subjects surveyed; the convenience sample may not necessarily be representative of the population living in Long Island/Queens/Staten Island.

## Conclusions

Despite these limitations, this is the first study of this kind conducted in the greater New York area, contributing the first results on individual responses to the utility of biobanks as sources of biologic material from the general population. While respondents were generally inclined to approve of genetic research, and those who disapproved did not do so based on an underlying moral objection to such research, there is a disconnect between the belief in the importance of genetic research and the willingness of individuals to participate themselves. This indicates a continued concern for the ways in which genetic materials are safeguarded once they are collected. Also indicated was a general lack of understanding about the various consent processes that go along with genetic research, which should be addressed further to ensure the successful continuation of biobanks.

## Competing interests

The authors have no competing interests.

## Authors’ contributions

SMK, MK and NN performed the data analysis, GK and PG developed the study design and protocol, MK, JW and FB conducted the surveys, SMK, IS and ET interpreted the results and wrote the manuscript. All authors read and approved the final manuscript.

## Pre-publication history

The pre-publication history for this paper can be accessed here:

http://www.biomedcentral.com/1471-2458/13/114/prepub
